# Bibliometric Research in Neurosurgery: A Review of the 50 Most Cited Bibliometric Publications in the Neurosurgical Literature

**DOI:** 10.7759/cureus.67247

**Published:** 2024-08-19

**Authors:** Abdulhakim B Jamjoom, Abdulhadi Y Gahtani, Jude M Jamjoom, Belal M Sharab, Yousuf K Khogeer, Omar M Jamjoom, Moajeb T Alzahrani

**Affiliations:** 1 Section of Neurosurgery, College of Medicine, King Saud Bin Abdulaziz University for Health Sciences, Jeddah, SAU; 2 College of Medicine, Alfaisal University, Riyadh, SAU; 3 College of Medicine, Ankara Yıldırım Beyazıt University, Ankara, TUR; 4 School of Medicine, University of Central Lancashire, Preston, GBR; 5 Department of Pharmaceutical Care Services, King Abdulaziz Medical City, Jeddah, SAU

**Keywords:** citation rates, publication trends, most influential, h-index, neurosurgery, science mapping, performance analysis, bibliometric research

## Abstract

Bibliometry is a popular research method that is used to explore and analyze large volumes of data in an effort to highlight trends, patterns, and impacts within a specific field. This review aimed at highlighting the characteristics and citation patterns of the high-impact bibliometric research studies that were published in the neurosurgical literature. Using PubMed and Google Scholar, the 50 (52 due to identical citation numbers for the lowest three articles) most cited bibliometric research publications were identified and reviewed. Information relating to the articles’ publication and bibliometric features were retrieved. The articles’ citation numbers were collected. The median article age and journal impact factor (IF) were eight years and 2.76, respectively. The majority of studies were published in World Neurosurgery and the Journal of Neurosurgery, which were the publishing journals for 18 (35%) and 12 (23%) articles, respectively. Twenty-six (50%) articles were first authored by researchers from the United States of America (USA). The highest bibliometric component was science mapping, which was the theme in 30 (58%) articles. The majority of the bibliometric focus was clinical topics/fields (22 (42%) articles) and neurosurgeons/departments (21 (40%) articles). The most popular bibliometric metric was the *h*-index (±variants), which was employed in 22 (42%) articles. The median size of analyzed data was 188, and the most frequently utilized databases were Scopus (22 (42%) articles) and Web of Science (21 (40%) articles). The median (range) citation numbers were 52 (29-238). The citation analysis showed significantly higher citation numbers for older articles (aged ≥ 8 years) and studies published in the Journal of Neurosurgery. The citation rates were not influenced by the size of the data, the searched databases, or the bibliometric features. In conclusion, the most cited bibliometric research publications in the neurosurgical literature were predominantly descriptive analyses of clinical topics/fields and performance analyses of neurosurgeons/departments. Their citation numbers were relatively modest and were positively influenced by the publication’s age and by a specific publishing journal but not by the bibliometric features of the study. Bibliometric research provides useful analytic tools that can be utilized in review studies and other practical purposes such as scholarly practices and policy decision-making.

## Introduction and background

Bibliometric research encompasses a set of validated statistical methods that are used to analyze the literature in order to explore trends, patterns, and impacts within a specific field. Compared to a systematic review that summarizes and combines the findings of the existing literature on a specific research topic, bibliometric analysis sums up large quantities of data to describe the state of intellectual structure and emerging trends of a study field [1,2]. Bibliometric practices have proved valuable across a wide range of disciplines, including medicine, science and technology, social sciences, education, and business and management [1-3]. Researchers use bibliometric analysis for a variety of reasons, such as detecting changing shifts in journal performance, collaboration patterns, and research elements [3]. The real value of bibliometric assessment remains in its capacity to process, categorize, explore, and report complex data. Additionally, it has the ability to present a network of ideas and topics in meaningful ways that enable researchers to identify knowledge gaps, derive novel concepts for investigation, and place their intended contributions to the field [1-3].

Bibliometric tools are quantitative by nature; however, they can be used to make statements about qualitative features. In fact, it has been suggested that the main purpose of bibliometric reviews is to transform something unquantifiable (scientific quality) into an assessable entity [4]. Bibliometric techniques can easily be scaled from micro (institute) to macro (world), and the evaluation of research can be carried out at the level of the journal, researcher, department, medical specialty, country, and worldwide regions [4,5]. The fundamental components of bibliometric evaluation are performance analysis and science mapping [1,2]. Performance analysis focuses on the appraisal of the output of research in a given field. It involves the use of publication- and citation-related metrics for the assessment of research sources (articles, journals), domains (subject fields), and contributors (authors, institutions, countries) [1,2]. Science mapping concentrates on the intellectual interactions and structural connections among research constituents. It makes it feasible to uncover the key matters along with the salient trends and gaps while shedding light on new developments in the field [1,2].

Citation analysis is an important basic technique in science mapping that functions on the assumption that citations reflect intellectual links between publications that develop when one publication cites the other [2,6]. Citation count might not be a criterion for quality assessment; nevertheless, articles with higher citation numbers are considered a milestone in any field and can affect the research and clinical approach. Furthermore, it is recognized that an article’s citation number will affect the publishing journal’s impact factor (IF) and can be regarded as reflective of the article's endorsement, efficacy, quality, and the author’s reputation [6]. Citation analysis allows researchers to identify the most cited publications in their field. Assessment of the most influential publications in any subject will enhance knowledge of research evolution and highlight subjects of relevance in that area. Evaluation of high-impact studies in specialties, subspecialties, journals, clinical topics, and research types has been a matter of interest that received attention in recent years [7-12]. Lately, bibliometric evaluation of systematic reviews and metanalyses has been a focus of several publications [9,11,12]. However, bibliometric assessment of bibliometric studies remains a topic that is limited to a few reports in the literature [6,13]. The purpose of this review is to identify and analyze the most cited bibliometric research studies that were published in neurosurgical literature. The study aimed to highlight the characteristics of bibliometric studies in the field of neurosurgery and to determine the factors that affect the citations among the 50 most influential articles on the subject.

## Review

Methods

Search Strategy

This study was carried out at King Saud Bin Abdulaziz University for Health Science, Jeddah, Kingdom of Saudi Arabia. No ethical approval was necessary by our institution as the study was based on data obtained from open-access sources. The PubMed database was searched on 15th December 2023 for suitable articles using the following combinations: (Title) Bibliometric OR Bibliometrics OR Cited OR Citation OR Citations OR Productivity OR Output OR Index OR Indices OR Level of Evidence OR Rank OR Ranking OR Rankings AND (Journal) Individual by name. The list of neurosurgical and spine journals searched and the number of screened articles are shown in Table 1.

**Table 1:** List of the searched neurosurgical and spine journals

Journals	Screened Articles	Bibliometric Articles	Most Cited Articles
World Neurosurgery	249	129	18
Journal of Neurosurgery	114	22	12
Spine	200	23	6
European Spine Journal	138	8	4
Spine Journal	73	4	3
Neurosurgery	98	9	2
Journal of Neurosurgery Spine	33	2	2
Child's Nervous System	36	14	2
Neurosurgical Review	27	10	1
Journal of Neurosurgery Pediatrics	21	7	1
Stere and Functional Neurosurgery	6	3	1
Clinical Neurology and Neurosurgery	54	8	0
Acta Neurochirurgica	49	5	0
Journal of Neurology Neurosurgery and Psychiatry	46	0	0
Spinal Cord	31	1	0
Joint Bone Spine	25	0	0
British Journal of Neurosurgery	23	8	0
Surgical Neurology International	22	11	0
Neurospine	17	3	0
Neurosurgical Focus	13	2	0
Neurologia Medico-Chirurgica	11	0	0
Journal of Neurosurgical Sciences	10	0	0
Journal of Korean Neurosurgical Society	10	2	0
Surgical Neurology	9	1	0
Asian Journal of Neurosurgery	9	2	0
Pediatric Neurosurgery	8	1	0
Pituitary	7	0	0
Journal of Neurological Surgery Part A Central European Neurosurgery	6	2	0
Journal Neurological Surgery Part B Skull Base	3	0	0
Clinical Neurosurgery	0	0	0
Total	1348	277	52

A bibliometric publication was defined as being one of the following [1,2]: (1) studies in which quantitative techniques were applied to bibliographic data, such as publications and citation metrics; (2) studies that evaluated performance whether at the levels of individuals, institutions, countries, subject areas, or journals; and (3) studies that analyzed the most influential publications and those that looked at trends, relationships among citing publications, topics, authors [1,2]. The search yielded a total of 1,348 studies, of which 1,071 were excluded due to being duplicates, non-bibliometric, or not providing adequate data.

**Figure 1:**
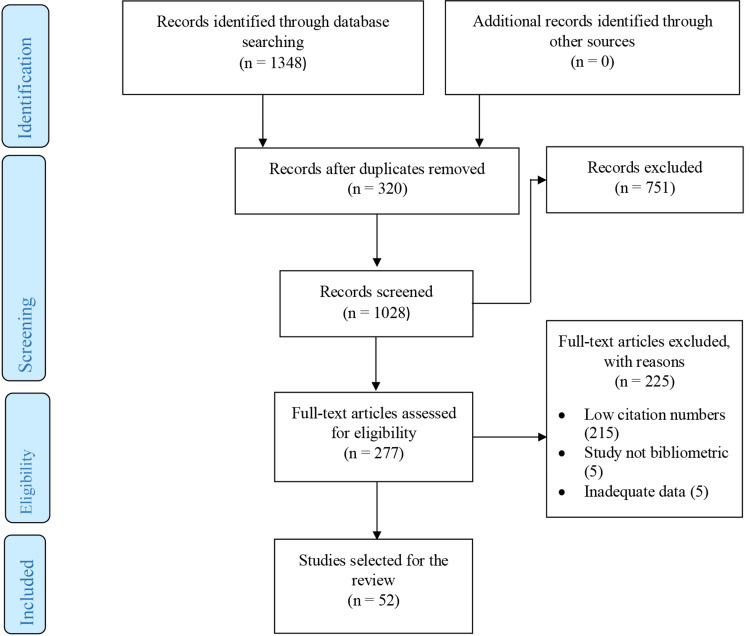
PRISMA flowchart for the review of the most cited bibliometric research publications in the neurosurgical literature PRISMA: Preferred Reporting Items for Systematic Reviews and Meta-Analyses

Using Google Scholar, the citation numbers for the remaining 277 articles were documented. In view of the regular changes in the citation numbers, the findings on a single day (30th March 2024) were recorded and used for analysis. The 50 most cited articles (52 because of identical citation numbers for the lower three articles) were identified and chosen for this review. The selection was limited to bibliometric research studies published in the neurosurgical and spine journals, which will be referred to hereinafter as neurosurgical journals or neurosurgical literature in this article. A Preferred Reporting Items for Systematic Reviews and Meta-Analyses (PRISMA) flow diagram showing the flow of the review phases is presented in Figure 1.

Analysis of Articles' Characteristics

Using the full articles, information relating to each of the selected studies was collected by two of the authors independently, and any discrepancies were resolved by consensus. The following data was collected: Article publication features: publishing year, journal and its IF, number of authors, number of centers, number of specialties, number of countries, number of references, and the first authors’ countries. Articles bibliometric features: bibliometric component (performance analysis, science mapping), bibliometric focus (neurosurgeons/departments, journals, clinical topics/ fields), bibliometric metrics (*h*-index (± variants), most influential studies, publication trends), data source (neurosurgical, medical journals), searched databases, analyzed data size, and the reporting of at least one significant finding. Missing data were referred to as not available (NA). The journals' IF data were obtained from an online source [14].

Analysis of Articles' Citation Patterns

The citation predictors assessment was carried out by correlating the citation numbers for the selected articles with the various articles' characteristics. The correlation testing was done by calculating the Pearson correlation coefficient (R) using the Social Sciences Statistics website [15], and significance was determined when P ≤ 0.05. A secondary citation analysis was carried out by comparing the mean citation numbers (± standard deviation (SD)) between the different subgroups. The median was taken as a cut-off point in the numerical parameters as follows: articles’ ages (≤ 8 versus (vs.) > 8 years), journals’ IF (≥ 2.75 vs. < 2.75), number of authors (> 4 vs. ≤ 4), number of centers (1 vs. > 1), number of specialties (1 vs. > 1), number of countries (1 vs. > 1), number of references (< 29 vs. ≥ 29), first authors’ countries (USA vs. others), bibliometric component (performance analysis vs. science mapping), bibliometric focus ((clinical topics/ fields vs. others), (neurosurgeons/ departments vs. others), (journals vs. others)), bibliometric metrics ((*h*-index vs. others), (most influential vs. others), (publication trends vs. others)), searched journals (medical vs. neurosurgical), searched databases (one database vs. > 1 database), analyzed data size (≥ 188 vs. < 188), and reporting of significant findings (yes vs. no). The statistical analysis was carried out by calculating the mean difference (MD) using the MedCalc website (https://www.mdcalc.com/) [16]. Significance was determined when P ≤ 0.05.

Results

The 52 most cited bibliometric research studies in the neurosurgical literature are summarized in Table 2 [17-68].

**Table 2:** Analysis of the 52 most cited bibliometric research studies published in the neurosurgical literature Abbreviations: NA: not available, G. Scholar: Google Scholar, NLM: National Library of Medicine, NIHRP: National Institute of Health Research Portfolio, WOS: Web of Science, SJR: Scimago Journal & Country Rank, AANS: American Association of Neurological Surgeons, J: Journal, Stereo: Stereotactic, Funct: Functional, Neurosurg: Neurosurgery, Nerv: Nervous, Syst: System, Pediatr: Pediatrics, SAH: subarachnoid hemorrhage

Rank	First Author (Year) [Ref]	Journal	Searched Databases	Bibliometric Focus	Bibliometric Metric	Data Size	Cites
1	Lee et al. (2009) [17]	Journal of Neurosurgery	G. Scholar & Scopus	Neurosurgeons	*h*-index (± variants)	30	238
2	Xie et al. (2020) [18]	World Neurosurgery	WOS	Atlanto-axial spine surgery	Most influential publications	3161	234
3	Ponce et al. (2010) [19]	Journal of Neurosurgery	WOS & Journal Citation Report	Neurosurgery journals	Most influential publications	100	225
4	Khan et al. (2014) [20]	Journal of Neurosurgery	WOS & Scopus & G. Scholar	Neurosurgeons & departments	*h*-index (± variants)	1225	143
5	Murray et al. (2012) [21]	European Spine J	WOS	Spine journals	Most influential publications	100	137
6	Spearman et al. (2010) [22]	Journal of Neurosurgery	G. Scholar	Neurosurgeons	*h* index (± variants)	1120	108
7	Ponce et al. (2010) [23]	Journal of Neurosurgery	WOS	Departments	*h* index (± variants)	113	91
8	Akmal et al. (2020) [24]	World Neurosurgery	Scopus	Glioblastoma multiforme	Most influential publications	100	87
9	Aoun et al. (2013) [25]	World Neurosurgery	NA	Neurosurgeons	*h*-index (± variants)	NA	85
10	Ponce et al. (2010) [26]	Journal of Neurosurgery	WOS	Neurosurgery journals	Most influential publications	106	81
11	Hauptman et al. (2011) [27]	Journal of Neurosurgery	MEDLINE	Global productivity, focus & funding	Publication trends	53,425	80
12	Venable et al. (2014) [28]	World Neurosurgery	G. Scholar & Scopus & NIHRP	Neurosurgeons & departments	*h*-index (± variants)	1225	77
13	Wei et al. (2016) [29]	European Spine Journal	Scopus	Spine surgery	Publication trends	13,115	76
14	Guo et al. (2019) [30]	World Neurosurgery	WOS	Stem cell in spinal cord injury	Publication trends	4188	73
15	Yuen et al. (2018) [31]	World Neurosurgery	Scopus & WOS & NLM	Neurosurgery & spine journals	*h*-index (± variants)	54	69
16	Alotaibi et al. (2016) [32]	World Neurosurgery	SJR portal	Departments & journals	*h*-index (± variants)	36	62
17	Khan et al. (2013) [33]	World Neurosurgery	Scopus & G. Scholar	Neurosurgeons	*h*-index (± variants)	188	62
18	Sarkiss et al. (2017) [34]	Neurosurgery	PubMed & Scopus	Neurosurgeons (residents)	*h*-index (± variants)	1325	61
19	Lin et al. (2020) [35]	European Spine J	WOS	Full endoscopic spine surgery	Publication trends	408	61
20	Khan et al. (2019) [36]	Neurosurgery	Scopus	Neurosurgeons (residents)	*h*-index (± variants)	1506	57
21	Khan et al. (2013) [37]	World Neurosurgery	Scopus	Neurosurgeons & departments	*h*-index (± variants)	188	56
22	Alotaibi et al. (2016) [38]	World Neurosurgery	G. Scholar	Aneurysmal SAH	Most influential publications	100	55
23	Agarwal et al. (2013) [39]	World Neurosurgery	Scopus	Neurosurgeons	*h*-index (±variants)	869	54
24	Kiraz et al. (2020) [40]	World Neurosurgery	WOS	Spinal cord injury	Publication trends	13,662	53
25	Fan et al. (2017) [41]	Spine	WOS	Minimally invasive spine	Publication trends	2051	52
26	Huang et al. (2020) [42]	Spine	WOS	Sacral fracture surgery	Publication trends	611	52
27	Rothoerl et al. (2003) [43]	Neurosurgical Review	WOS	Neurosurgery journals	Publication trends	982	51
28	Wupperman et al. (2007) [44]	Spine	NA	Spine Journals	Publication trends	112	50
29	De la Garza-Ramos et al. (2016) [45]	J Neurosurg Spine	WOS	Spinal oncology	Most influential publications	100	48
30	Wilkes et al. (2015) [46]	Journal of Neurosurgery	Scopus	Neurosurgeons & departments	*h*-index (± variants)	315	48
31	Campbell et al. (2011) [47]	Journal of Neurosurgery	Scopus & WOS	Neurosurgeons & departments	*h*-index (± variants)	986	48
32	Klimo et al. (2014) [48]	J Neurosur Pediatr	Scopus & G. Scholar	Neurosurgeons	*h*-index (± variants)	312	45
33	Kashkoush et al. (2017) [49]	World Neurosurgery	Scopus	Neurosurgeons (residents)	*h*-index (± variants)	206	44
34	Badhiwala et al. (2018) [50]	Spine	WOS	Spinal disorders	Most influential publications	100	42
35	Reddy et al. (2020) [51]	Journal of Neurosurgery	iCite database	Neurosurgeons	*h*-index (± variants)	1687	42
36	Almutairi et al. (2017) [52]	World Neurosurgery	Scopus	Meningioma	Most influential publications	100	41
37	Andrade et al. (2013) [53]	Spine Journal	PubMed & WOS	Surgery for chronic back pain	Publication trends	39	41
38	Taylor et al. (2015) [54]	Journal of Neurosurgery	Scopus	Departments	*h*-index (± variants)	103	40
39	Schoenfeld et al. (2015) [55]	Spine Journal	Scopus & PubMed	Neurosurgeons	*h* index (± variants)	282	39
40	Chen et al. (2019) [56]	J Neurosurg Spine	PubMed	Cervical myelopathy	Publication trends	1008	38
41	Venable et al. (2014) [57]	Child's Nerv Syst	Scopus	Pediatric neurosurgery	Publication trends	25	37
42	Agarwal et al. (2020) [58]	World Neurosurgery	AANS Medical Students Chapters	Medical students interest groups	Publication trends	121	37
43	Lipsman et al. (2012) [59]	Stereo Funct Neurosurgery	G. Scholar	Stereotactic and functional	Most influential publications	100	35
44	Huang et al. (2016) [60]	Spine	WOS	Back pain research	Most influential publications	100	35
45	Jamjoom et al. (2016) [61]	World Neurosurgery	Scopus & G. Scholar	Neurosurgeons	*h*-index (±variants)	317	34
46	Brinker et al. (2018) [62]	Spine	PubMed	Spine Journals	Publication trends	1566	34
47	Wilcox et al. (2013) [63]	Child's Nerv System	WOS & Journal Citation Reports	Pediatric neurosurgery	Most influential publications	100	33
48	Amiri et al. (2013) [64]	Spine Journal	NA	Spine Journals	Publication trends	703	32
49	Khan et al. (2015) [65]	World Neurosurgery	Scopus & WOS	Skull base neurosurgery	Most influential publications	100	30
50	Guo et al. (2018) [66]	World Neurosurgery	WOS	Pituitary adenoma	Most influential publications	100	29
51	Nowrouzi et al. (2017) [67]	European Spine J	Publish or Perish	Spinal cord injury	Most influential publications	50	29
52	Lozano et al. (2015) [68]	Journal of Neurosurgery	Scopus	Departments	*h*-index (±variants)	1217	29

Articles Publication Features

**Figure 2:**
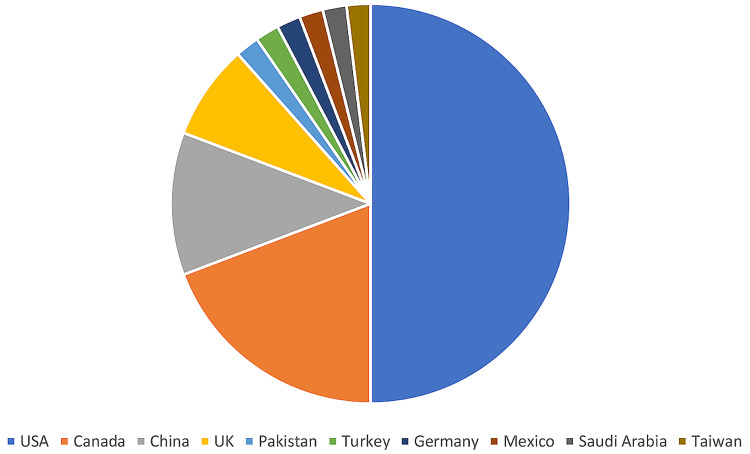
Pie chart showing the distribution of the 52 most cited bibliometric publications in the neurosurgical literature according to the first authors’ countries

The median (range) publication year and articles’ age were 2015 (2003-2020) and eight (3-20) years, respectively. The publishing journals are listed in Table 1. The most common journals and number of articles were World Neurosurgery: 18 (35%), Journal of Neurosurgery: 12 (23%), Spine: six (12%), European Spine Journal: four (7%), and Spine Journal: three (6%). The median (range) journals’ IF was 2.76 (1.53-5.32). The median (range) number of authors was 4 (1-20). The median (range) number of centers was 1 (1-13). The median (range) number of specialties was 1 (1-5). The median (range) number of countries was 1 (1-5), and the median (range) number of references was 29 (11-124). The distribution of articles according to the first authors’ countries is shown in Figure 2. The countries and number of articles were USA: 26 (50%), Canada: 10 (19%), China: six (12%), UK: four (8%), and others: six (12%).

Articles Bibliometric Features

**Figure 3:**
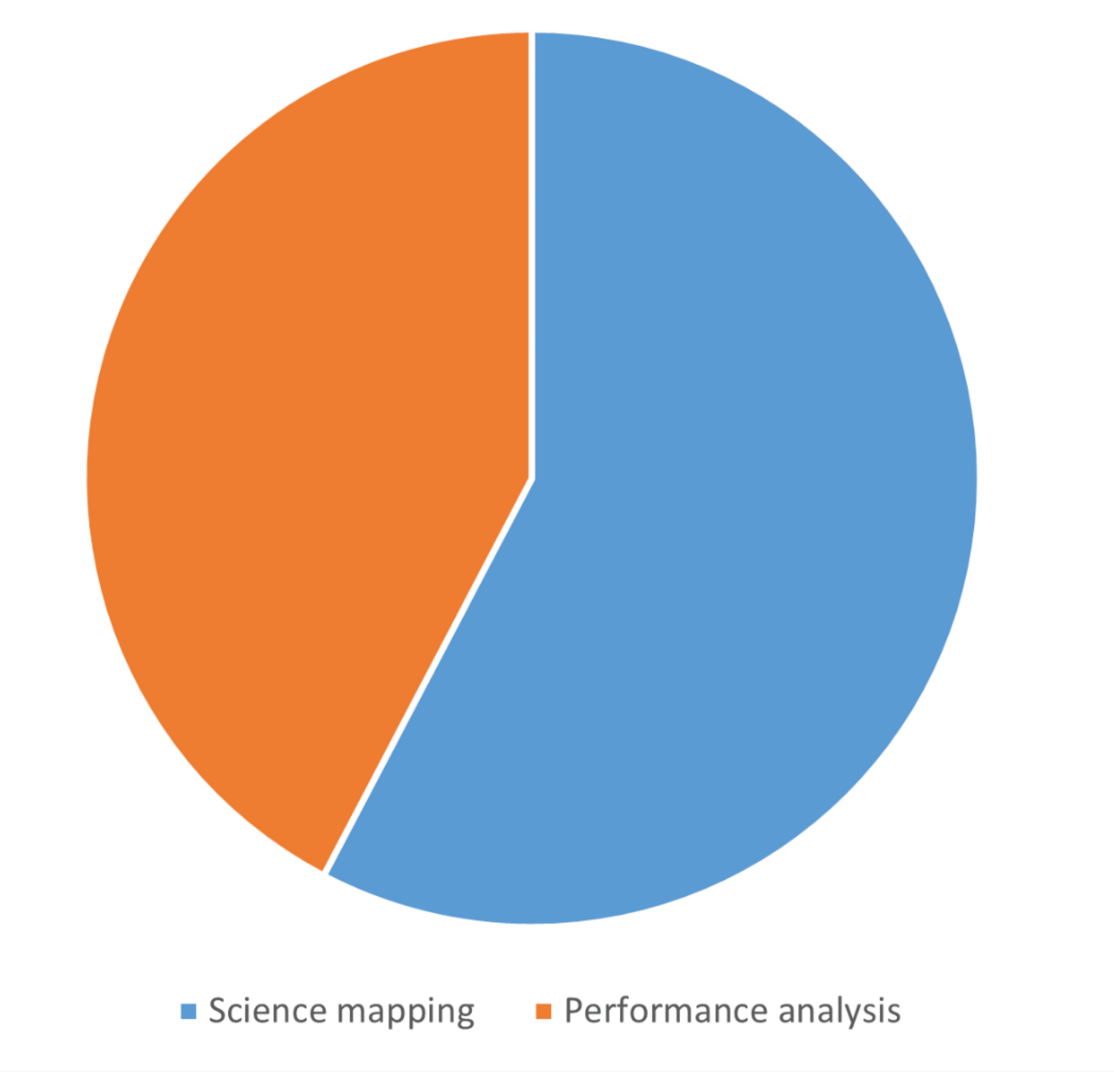
Pie chart showing the distribution of the 52 most cited bibliometric publications in the neurosurgical literature according to the two bibliometric components

**Figure 4:**
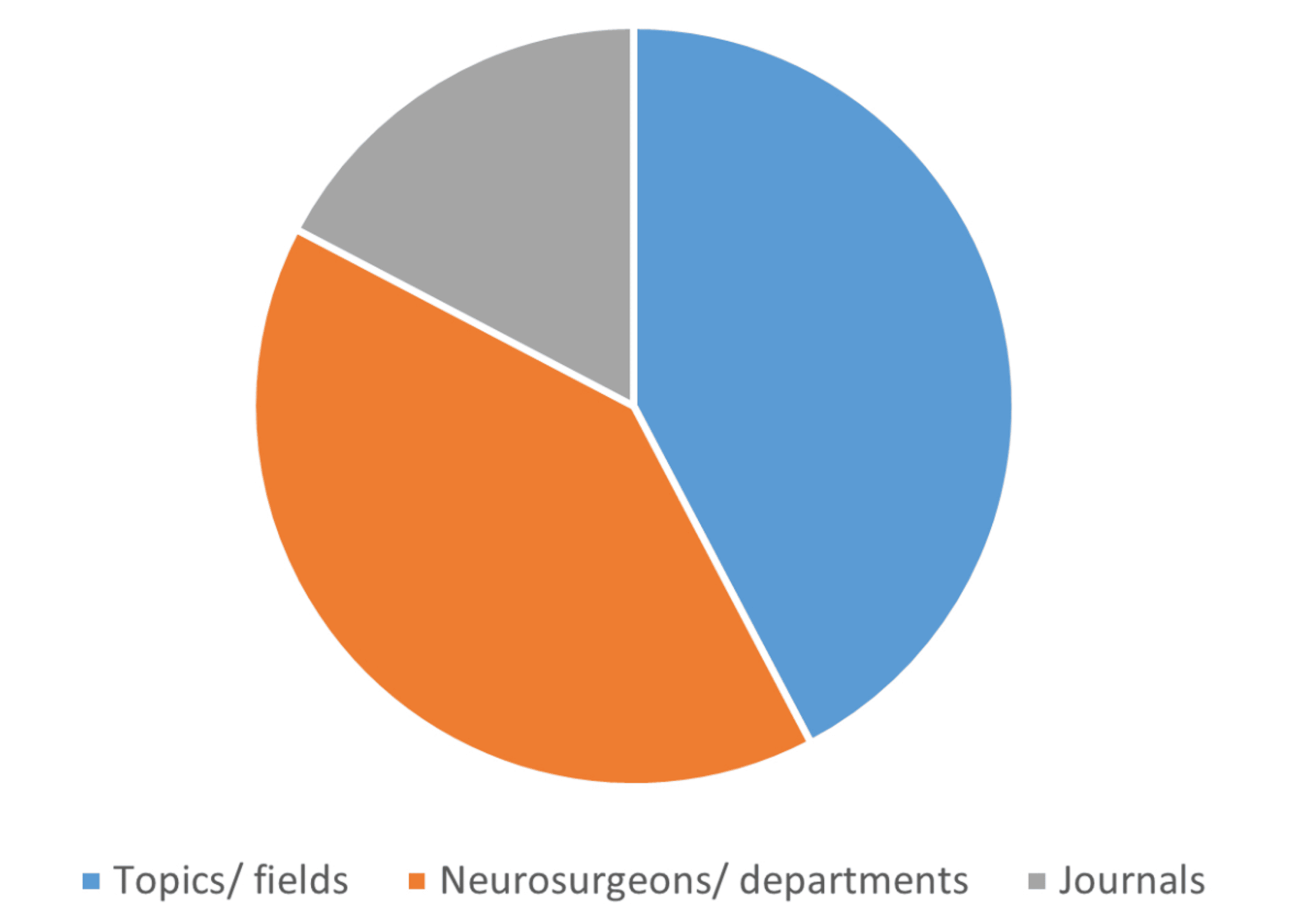
Pie chart showing the distribution of the 52 most cited bibliometric publications in the neurosurgical literature according to the three bibliometric focuses

**Figure 5:**
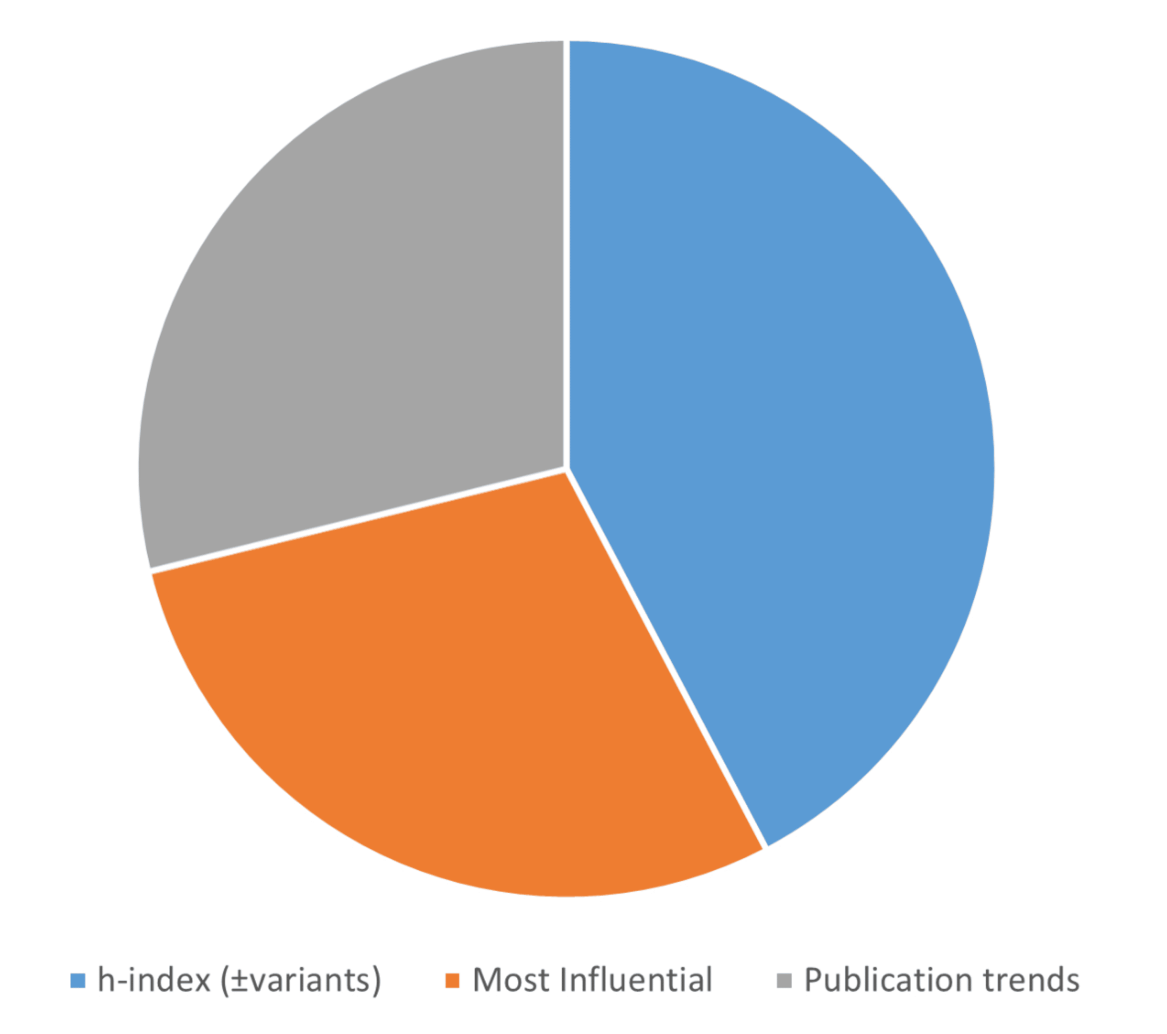
Pie chart showing the distribution of the 52 most cited bibliometric publications in the neurosurgical literature according to the three bibliometric metrics

The distribution of the articles according to their bibliometric components, focuses, and metrics is illustrated in Figures 3-5. The number of articles based on the bibliometric component was science mapping: 30 (58%) and performance analysis: 22 (42%). The number of articles based on the bibliometric focus was clinical topics/fields: 22 (42%), neurosurgeons/departments: 21 (40%), and neurosurgical journals: nine (17%). The number of articles based on the bibliometric metric was *h*-index (± variants): 22 (42%), most influential studies: 15 (29%), and publication trends: 15 (29%). The number of articles according to data sources was medical journals: 44 (85%) and neurosurgical journals: eight (15%). The median (range) analyzed data size was 188 (30-53425). The most commonly utilized databases and number of articles were Scopus: 22 (42%), Web of Science: 21 (40%), Google Scholar: nine (17%), PubMed: five (10%), Journal Citation Report: two (4%), and others: six (12%). A report of at least one significant finding was found in 30 (58%) articles.

Articles Citation Patterns

The median (range) article citation numbers were 52 (29-238). Tables 3-4 summarise the correlation and secondary analysis findings between the citation numbers and the various articles' characteristics. The correlation analysis showed no significant association between citation numbers and any of the publication and bibliometric features. The secondary analysis, however, demonstrated significantly higher mean citation numbers amongst older articles (aged ≥ 8 years) (P=0.0392) and in studies published in the Journal of Neurosurgery (P=0.0085). None of the other parameters reached significance.

**Table 3:** Summary of the correlation analysis between the citation numbers and the various articles characteristics for the 52 most cited bibliometric research publications in the neurosurgical literature None of the findings reached significance at P ≤ 0.05

Features	R-Value	P-Value
Articles’ age in years	0.2364	0.0916
Articles’ publishing journals	0.2396	0.0871
Articles’ journals IF	0.1818	0.197
Bibliometric components	0.0623	0.6608
Bibliometric focuses	0.0537	0.7054
Bibliometric metrics	0.1456	0.3031
Searched journals	0.0281	0.8432
Searched databases	0.2105	0.1466
Analysed data sizes	0.0586	0.6829
Report of one or more significant findings	0.0012	0.9933
Articles’ number of Authors	0.1968	0.1620
Articles’ number of centres	0.1148	0.4177
Articles’ number of specialties	0.0542	0.7027
Articles’ number of countries	0.075	0.5972
Articles’ number of references	0.1717	0.2236
Articles’ first author’s country	0.0711	0.6165

**Table 4:** Summary of the secondary mean difference analysis between the citation numbers and the various characteristics for the 52 most cited bibliometric research publications in the neurosurgical literature Abbreviations: *Data not available in some studies, **significant (P ≤ 0.05), N: Total number, IF: Impact Factor, depart: Departments

Articles Parameters	Variables	Number (N=52)	Total Cites (N=3440)	Mean Cite Numbers (±SD)	Mean Difference	P-value
Articles’ age in years	>8	22	1809	82.2±57.5	27.8	0.0392**
	≤8	30	1631	54.4±37.1		
Articles’ journals IF	≥2.75	26	1805	69.4±54.3	6.5	0.6326
	<2.75	26	1634	62.9±42.4		
Articles’ Publishing journals	Journal of Neurosurgery	12	1173	97.8±70.6	41	0.0085**
	Others	40	2267	56.7±35.4		
	World Neurosurgery	18	1182	65.7±45.6	0.7	0.9610
	Others	34	2258	66.4±50.4		
Bibliometric components	Performance analysis	22	1532	69.6±46	6	0.6631
	Science mapping	30	1908	63.6±50.7		
Bibliometric focuses	Topics/fields	22	1261	57.3±42	19.3	0.1571
	Others	30	2179	72.6±51.7		
	Neurosurgeons/dept	21	1438	68.5±47.6	3.9	0.7786
	Others	31	2002	64.6±49.6		
	Journals	9	741	82.3±62.1	19.5	0.2760
	Others	43	2699	62.8±45.2		
Bibliometric metrics	h-index (± variants)	22	1532	69.6±46	6	0.6631
	Others	30	1908	63.6±50.7		
	Most influential	15	1141	76.1±68.9	14	0.3497
	Others	37	2299	62.1±37.6		
	Publication trends	15	767	51.1±15.5	21	0.1562
	Others	37	2673	72.2±55.6		
Searched journals	Medical journals	44	2936	66.7±45.5	3.7	0.8446
	Neurosurgery	8	504	63±65.9		
Searched databases*	One database	35	1145	60.8±38.4	21	0.1823
	> 1 database	14	2128	81.8±69.6		
Analysed data size*	≥ 188	28	1783	63.7±41.1	4.7	0.7357
	< 188	23	1572	68.4±57.6		
Report of sig. findings*	One or more	30	1975	65.8±40.6	0.1	0.9943
	None	21	1380	65.7±59.7		
Articles number of authors	≤4	21	1652	78.7±57.8	21	0.1258
	>4	31	1788	57.7±39.6		
Articles number of centres	1	18	1403	77.9±63.5	18	0.2047
	>1	34	2037	59.9±37.7		
Articles number of specialties	1	35	2407	68.8±56.4	8	0.5811
	>1	17	1033	60.8±25.7		
Articles number of countries	1	43	2689	62.5±46.6	20.9	0.2423
	>1	9	751	83.4± 55.8		
Articles number of references	<29	26	1943	74.7±60.9	17.1	0.2055
	≥29	26	1497	57.6±30.2		
Articles first author’s country	USA	24	1593	66.4±47.6	0.4	0.9766
	Others	28	1847	66±49.9		

Discussion

Reputable journals and researchers have published highly cited articles utilizing bibliometric methods to explore the progress and emerging trends in various specialties. For bibliometric research to be useful and contribute to advancing theory and practice, it is essential to be of high quality [1,2]. Mukherjee et al. [1] identified seven factors that are useful in developing and evaluating effective bibliometric research. These are the following: novelty (what’s new?), value (so what?), importance (who cares?), timeliness (why now?), exposition (why so?), rigor (well done?), and completeness (done well?). Furthermore, for the reporting of bibliometric research to be judged sound, several parameters should be mentioned clearly in the article. These include clear objectives, comprehensive systematic search using multiple databases with well-defined inclusion and exclusion criteria, suitable use of bibliometric indicators, proper checking of data for accuracy and normalization, appropriate data analysis utilizing advanced techniques and software tools, clear and informative visualization, and reasonable contextualization of findings [1-3].

Amongst the 52 most cited bibliometric studies in the neurosurgical literature, the *h*-index (± variants) was utilized in the 22 articles that were categorized as performance analysis. The metric measurement in these studies was calculated for neurosurgeons in nine articles [17,22,25,33,39,48,51,55,61], for neurosurgeons and departments in five articles [20,28,37,46,47], for departments in three articles [23,54,68], for neurosurgical residents in three articles [34,36,49], and for neurosurgical and spine journals in two articles [31,32]. Apart from the latter and one review article [25], the data pool was neurosurgeons and departments from the USA in 12 articles [17,20,22,28,33,34,37,39,47,49,41,54], from the USA and Canada in four articles [23,36,48,55], from the UK in two articles [46,61], and from Canada in one article [68].

The 30 articles that were categorized as science mapping included equal numbers of the most influential and publication trends papers. These articles cover a broad spectrum of topics in neurosurgery. Of the 15 most influential publications, three articles looked at publications in neurosurgical and spine journals in general [19,21,26]. The others concentrated on a specific clinical entity or a subspecialty. The variety of the areas covered included atlanto-axial spine surgery [18], glioblastoma multiforme [24], aneurysmal subarachnoid hemorrhage [38], spinal disorders [50], meningioma [52], back pain research [60], pituitary adenoma [66], spinal cord injury [67], spinal oncology [45], pediatric neurosurgery [63], stereotactic and functional neurosurgery [59], and skull base surgery [65]. Of the 15 publication trends studies, three analyzed the level of evidence of publications in neurosurgical and spine journals [43,44,64]. The remaining examined trends in productivity relating to a specific issue, subspecialty, or journal. The range of subjects covered included globalization, focus, and funding [27]; spine surgery [29]; stem cell therapy for spinal cord injury [30]; full endoscopic spine surgery [35]; spinal cord injury [40]; minimally invasive spine surgery [41]; sacral fracture surgery [42]; back pain surgery [53]; cervical myelopathy [56]; pediatric neurosurgery journals [57]; medical students interest groups [58]; and gender and collaboration impact on authorship [62].

The median citation number for the most cited bibliometric research studies published in the neurosurgical literature was 52 citations. This was lower than the citation numbers for higher levels of evidence research studies such as the top 100 trials on glioblastoma multiforme (median 349 citations) [7]. It was also lower than the citation numbers for the top 50 survey research publications in the neurosurgical literature (median 111 citations) [8]. Variation in citation rates according to study design and subject is well recognized in the literature [69]. We found that the age of the publication (≥ 8 years) was a significant predictor of citation numbers. We also observed a positive link between citation rates and the bibliometric study being published in the Journal of Neurosurgery (IF = 4.41) [14]. The correlation between the publishing journals’ IF and citation numbers however was close but did not reach significance (P = 0.0871). The impact of the publishing journal’s IF on citation rates is well documented in the literature [69]. In this review, the association may have been influenced by the number and age of the articles that were published in the Journal of Neurosurgery in particular. The median data size was 188, and it ranged from 30 (neurosurgeons) [17] to 53,425 (articles) [27]. Unlike other studies that reported an association between sample size and citation numbers [8,69], no correlation between data size and citation rates was observed here. The most popular databases used included Scopus (42%), Web of Science (40%), Google Scholar (17%), and PubMed (10%). Furthermore, neither the choice of the database nor the use of more than one database appeared to have influenced citation rates. In this review of bibliometric research in neurosurgery, no link was established between citation rates and all the other parameters that were tested. These were the bibliometric component, bibliometric focus, bibliometric metrics, report of at least one significant finding, numbers of authors, centers, specialties, countries, references, and the first authors’ countries.

There are several limitations to this study. The study relied on the precision of online search engines PubMed and Google Scholar. The study did not include bibliometric research studies that were published outside the neurosurgical journals. The selection of the 52 most cited studies was based on their total citations at a certain point, which was likely to change relatively quickly. This could have influenced the inclusion or exclusion of a few of the lower-impact bibliometric studies. The wide duration from publication (17 years) had probably affected the citations of older studies. The quality of the bibliometric analysis was not examined. Additionally, the changing trends in the reporting of bibliometric data over the years were not addressed. There may have been errors in the data collection. There may have been discrepancies in the allocation of articles into the various bibliometric categories. Defining the affiliation based on the first author may not reflect all authors of multi-disciplinary papers.

## Conclusions

The most cited bibliometric research publications in the neurosurgical literature were predominantly a descriptive analysis of clinical topics/fields and a performance analysis of neurosurgeons/departments. The most common metric used was the *h*-index (± variants). The majority were published in two journals (World Neurosurgery and the Journal of Neurosurgery) and first authored by researchers from the USA. Their citation numbers were relatively modest and were positively influenced by the publication’s age and by a specific publishing journal but not by the bibliometric features of the study, the size of analyzed data, or the databases used. Bibliometric research provides useful analytic tools that can be utilized in review studies and other practical purposes such as scholarly practices and policy decision-making.

## References

[1] Mukherjee D, Lim WM, Kumar S, Donthu N (2022). Guidelines for advancing theory and practice through bibliometric research. J Bus Res.

[2] Donthu N, Kumar S, Mukherjee D, Pandey N, Lim WM (2021). How to conduct a bibliometric analysis: an overview and guidelines. J Bus Res.

[3] Lim WM, Kumar S (2024). Guidelines for interpreting the results of bibliometric analysis: a sensemaking approach. Glob Bus Organ Excell.

[4] Wallin JA (2005). Bibliometric methods: pitfalls and possibilities. Basic Clin Pharmacol Toxicol.

[5] Bardeesi AM, Jamjoom AA, Sharab MA, Jamjoom AB (2021). Clinical neuroscience research in Saudi Arabia: a bibliometric appraisal of productivity and worldwide ranking. Int J Med Res Health Sci.

[6] Daryakenari G, Batooli Z (2022). A bibliometric and subject analysis of 3300 most-cited articles in dentistry. Clin Exp Dent Res.

[7] Jamjoom AM, Gahtani AY, Jamjoom AB (2021). Predictors of citation rates in high-impact glioblastoma clinical trials. Cureus.

[8] Jamjoom AB, Gahtani AY, Jamjoom JM, Sharab BM, Jamjoom OM, AlZahrani MT (2024). Survey research among neurosurgeons: a bibliometric review of the characteristics, quality, and citation predictors of the top 50 most-influential publications in the neurosurgical literature. Cureus.

[9] Fu Y, Mao Y, Jiang S, Luo S, Chen X, Xiao W (2023). A bibliometric analysis of systematic reviews and meta-analyses in ophthalmology. Front Med (Lausanne).

[10] Ahmad SJ, Ahmed AR, Kowalewski KF (2020). Citation classics in general medical journals: assessing the quality of evidence; a systematic review. Gastroenterol Hepatol Bed Bench.

[11] Yang Y, Ma Y, Chen L, Liu Y, Zhang Y (2020). The 100 top-cited systematic reviews/meta-analyses on diabetic research. J Diabetes Res.

[12] Tarazona-Álvarez B, López-Roldán A, Vidal-Infer A, Alonso-Arroyo A (2023). Bibliometric study of the systematic reviews and meta-analyses in dentistry. J Clin Exp Dent.

[13] Gupta SM, Naqvi WM, Mutkure KN, Varma A, Thakur S, Umate R (2022). Bibliometric analysis on bibliometric studies of case reports in the medical field. Cureus.

[14] (2024). Journal impact. https://www.bioxbio.com/journal.

[15] (2024). Social science statistics. https://www.socscistatistics.com.

[16] (2024). User-friendly statistical software. https://www.medcalc.org/.

[17] Lee J, Kraus KL, Couldwell WT (2009). Use of the h index in neurosurgery. Clinical article. J Neurosurg.

[18] Xie L, Chen Z, Wang H, Zheng C, Jiang J (2020). Bibliometric and visualized analysis of scientific publications on atlantoaxial spine surgery based on Web of Science and VOSviewer. World Neurosurg.

[19] Ponce FA, Lozano AM (2010). Highly cited works in neurosurgery. Part I: the 100 top-cited papers in neurosurgical journals. J Neurosurg.

[20] Khan NR, Thompson CJ, Taylor DR, Venable GT, Wham RM, Michael LM 2nd, Klimo P Jr (2014). An analysis of publication productivity for 1225 academic neurosurgeons and 99 departments in the United States. J Neurosurg.

[21] Murray MR, Wang T, Schroeder GD, Hsu WK (2012). The 100 most cited spine articles. Eur Spine J.

[22] Spearman CM, Quigley MJ, Quigley MR, Wilberger JE (2010). Survey of the h index for all of academic neurosurgery: another power-law phenomenon?. J Neurosurg.

[23] Ponce FA, Lozano AM (2010). Academic impact and rankings of American and Canadian neurosurgical departments as assessed using the h index. J Neurosurg.

[24] Akmal M, Hasnain N, Rehan A (2020). Glioblastome multiforme: a bibliometric analysis. World Neurosurg.

[25] Aoun SG, Bendok BR, Rahme RJ, Dacey RG Jr, Batjer HH (2013). Standardizing the evaluation of scientific and academic performance in neurosurgery--critical review of the "h" index and its variants. World Neurosurg.

[26] Ponce FA, Lozano AM (2010). Highly cited works in neurosurgery. Part II: the citation classics. J Neurosurg.

[27] Hauptman JS, Chow DS, Martin NA, Itagaki MW (2011). Research productivity in neurosurgery: trends in globalization, scientific focus, and funding. J Neurosurg.

[28] Venable GT, Khan NR, Taylor DR, Thompson CJ, Michael LM, Klimo P Jr (2014). A correlation between National Institutes of Health funding and bibliometrics in neurosurgery. World Neurosurg.

[29] Wei M, Wang W, Zhuang Y (2016). Worldwide research productivity in the field of spine surgery: a 10-year bibliometric analysis. Eur Spine J.

[30] Guo S, Wang L, Xie Y (2019). Bibliometric and visualized analysis of stem cells therapy for spinal cord injury based on Web of Science and CiteSpace in the last 20 years. World Neurosurg.

[31] Yuen J (2018). Comparison of impact factor, eigenfactor metrics, and SCImago journal rank indicator and h-index for neurosurgical and spinal surgical journals. World Neurosurg.

[32] Alotaibi NM, Guha D, Fallah A (2016). Social media metrics and bibliometric profiles of neurosurgical departments and journals: is there a relationship?. World Neurosurg.

[33] Khan NR, Thompson CJ, Taylor DR, Gabrick KS, Choudhri AF, Boop FR, Klimo P Jr (2013). Part II: should the h-index be modified? An analysis of the m-quotient, contemporary h-index, authorship value, and impact factor. World Neurosurg.

[34] Sarkiss CA, Riley KJ, Hernandez CM, Oermann EK, Ladner TR, Bederson JB, Shrivastava RK (2017). Academic productivity of US neurosurgery residents as measured by h-index: program ranking with correlation to faculty productivity. Neurosurgery.

[35] Lin GX, Kotheeranurak V, Mahatthanatrakul A (2020). Worldwide research productivity in the field of full-endoscopic spine surgery: a bibliometric study. Eur Spine J.

[36] Khan NR, Saad H, Oravec CS (2019). An analysis of publication productivity during residency for 1506 neurosurgical residents and 117 residency departments in North America. Neurosurgery.

[37] Khan N, Thompson CJ, Choudhri AF, Boop FA, Klimo P Jr (2013). Part I: the application of the h-index to groups of individuals and departments in academic neurosurgery. World Neurosurg.

[38] Alotaibi NM, Nassiri F, Badhiwala JH, Witiw CD, Ibrahim GM, Macdonald RL, Lozano AM (2016). The most cited works in aneurysmal subarachnoid hemorrhage: a bibliometric analysis of the 100 most cited articles. World Neurosurg.

[39] Agarwal N, Clark S, Svider PF, Couldwell WT, Eloy JA, Liu JK (2013). Impact of fellowship training on research productivity in academic neurological surgery. World Neurosurg.

[40] Kiraz M, Demir E (2020). A bibliometric analysis of publications on spinal cord injury during 1980-2018. World Neurosurg.

[41] Fan G, Han R, Zhang H, He S, Chen Z (2017). Worldwide research productivity in the field of minimally invasive spine surgery: a 20-year survey of publication activities. Spine (Phila Pa 1976).

[42] Huang T, Wu H, Yang S (2020). Global trends of researches on sacral fracture surgery: a bibliometric study based on VOSviewer. Spine (Phila Pa 1976).

[43] Rothoerl RD, Klier J, Woertgen C, Brawanski A (2003). Level of evidence and citation index in current neurosurgical publications. Neurosurg Rev.

[44] Wupperman R, Davis R, Obremskey WT (2007). Level of evidence in Spine compared to other orthopedic journals. Spine (Phila Pa 1976).

[45] De la Garza-Ramos R, Benvenutti-Regato M, Caro-Osorio E (2016). The 100 most-cited articles in spinal oncology. J Neurosurg Spine.

[46] Wilkes FA, Akram H, Hyam JA, Kitchen ND, Hariz MI, Zrinzo L (2015). Publication productivity of neurosurgeons in Great Britain and Ireland. J Neurosurg.

[47] Campbell PG, Awe OO, Maltenfort MG, Moshfeghi DM, Leng T, Moshfeghi AA, Ratliff JK (2011). Medical school and residency influence on choice of an academic career and academic productivity among neurosurgery faculty in the United States. Clinical article. J Neurosurg.

[48] Klimo P Jr, Venable GT, Khan NR, Taylor DR, Shepherd BA, Thompson CJ, Selden NR (2014). Bibliometric evaluation of pediatric neurosurgery in North America. J Neurosurg Pediatr.

[49] Kashkoush A, Prabhu AV, Tonetti D, Agarwal N (2017). The neurosurgery match: a bibliometric analysis of 206 first-year residents. World Neurosurg.

[50] Badhiwala JH, Nassiri F, Witiw CD (2018). Highly cited works in spinal disorders: the top 100 most cited papers published in spine journals. Spine (Phila Pa 1976).

[51] Reddy V, Gupta A, White MD (2020). Assessment of the NIH-supported relative citation ratio as a measure of research productivity among 1687 academic neurological surgeons. J Neurosurg.

[52] Almutairi O, Albakr A, Al-Habib A, Ajlan A (2017). The top-100 most-cited articles on meningioma. World Neurosurg.

[53] Andrade NS, Flynn JP, Bartanusz V (2013). Twenty-year perspective of randomized controlled trials for surgery of chronic nonspecific low back pain: citation bias and tangential knowledge. Spine J.

[54] Taylor DR, Venable GT, Jones GM (2015). Five-year institutional bibliometric profiles for 103 US neurosurgical residency programs. J Neurosurg.

[55] Schoenfeld AJ, Bhalla A, George J, Harris MB, Bono CM (2015). Academic productivity and contributions to the literature among spine surgery fellowship faculty. Spine J.

[56] Chen YC, Kuo CH, Cheng CM, Wu JC (2019). Recent advances in the management of cervical spondylotic myelopathy: bibliometric analysis and surgical perspectives. J Neurosurg Spine.

[57] Venable GT, Shepherd BA, Roberts ML, Taylor DR, Khan NR, Klimo P Jr (2014). An application of Bradford's law: identification of the core journals of pediatric neurosurgery and a regional comparison of citation density. Childs Nerv Syst.

[58] Agarwal P, Khalafallah AM, Hersh EH, Ivan ME, Mukherjee D (2020). Impact of American Association of Neurological Surgeons medical student interest groups on participation in organized neurosurgery, research productivity, and residency match success. World Neurosurg.

[59] Lipsman N, Lozano AM (2012). Measuring impact in stereotactic and functional neurosurgery: an analysis of the top 100 most highly cited works and the citation classics in the field. Stereotact Funct Neurosurg.

[60] Huang W, Wang L, Wang B, Yu L, Yu X (2016). Top 100 cited articles on back pain research: a citation analysis. Spine (Phila Pa 1976).

[61] Jamjoom AA, Wiggins AN, Loan JJ, Emelifeoneu J, Fouyas IP, Brennan PM (2016). Academic productivity of neurosurgeons working in the United Kingdom: insights from the h-index and its variants. World Neurosurg.

[62] Brinker AR, Liao JL, Kraus KR (2018). Bibliometric analysis of gender authorship trends and collaboration dynamics over 30 years of spine 1985 to 2015. Spine (Phila Pa 1976).

[63] Wilcox MA, Khan NR, McAbee JH, Boop FA, Klimo P Jr (2013). Highly cited publications in pediatric neurosurgery. Childs Nerv Syst.

[64] Amiri AR, Kanesalingam K, Cro S, Casey AT (2013). Level of evidence of clinical spinal research and its correlation with journal impact factor. Spine J.

[65] Khan NR, Lee SL, Brown M (2015). Highly cited works in skull base neurosurgery. World Neurosurg.

[66] Guo X, Gao L, Wang Z, Feng C, Xing B (2018). Top 100 most-cited articles on pituitary adenoma: a bibliometric analysis. World Neurosurg.

[67] Nowrouzi B, Assan-Lebbe A, Sharma B, Casole J, Nowrouzi-Kia B (2017). Spinal cord injury: a review of the most-cited publications. Eur Spine J.

[68] Lozano CS, Tam J, Kulkarni AV, Lozano AM (2015). The academic productivity and impact of the University of Toronto Neurosurgery Program as assessed by manuscripts published and their number of citations. J Neurosurg.

[69] Tahamtan I, Safipour Afshar A, Ahamdzadeh K (2016). Factors affecting number of citations: a comprehensive review of the literature. Scientometrics.

